# The Roles of microRNA miR-185 in Digestive Tract Cancers

**DOI:** 10.3390/ncrna8050067

**Published:** 2022-10-08

**Authors:** Esmaeel Babaeenezhad, Fakhraddin Naghibalhossaini, Masoumeh Rajabibazl, Zohreh Jangravi, Forouzan Hadipour Moradi, Mohammad Davood Fattahi, Jörg D. Hoheisel, Mostafa Moradi Sarabi, Soroosh Shahryarhesami

**Affiliations:** 1 Student Research Committee, Baqiyatallah University of Medical Sciences, Tehran 1435916471, Iran; 2 Nutritional Health Research Center, Lorestan University of Medical Sciences, Khorramabad 6813816314, Iran; 3 Department of Clinical Biochemistry, School of Medicine, Student Research Committee, Shahid Beheshti University of Medical Sciences, Tehran 1983969411, Iran; 4 Department of Biochemistry, School of Medicine, Shiraz University of Medical Sciences, Shiraz 7134814336, Iran; 5 Department of Clinical Biochemistry, Faculty of Medicine, Shahid Beheshti University of Medical Sciences, Tehran 1983969411, Iran; 6 Department of Biochemistry, Faculty of Medicine, Baqiyatallah University of Medical Sciences, Tehran 1435916471, Iran; 7 Razi Herbal Medicines Research Center, Lorestan University of Medical Sciences, Khorramabad 6813816314, Iran; 8 Functional Genome Analysis, German Cancer Research Center (DKFZ), Im Neuenheimer Feld 580, 69120 Heidelberg, Germany; 9 Department of Biochemistry and Genetics, School of Medicine, Lorestan University of Medical Sciences, Khorramabad 6813816314, Iran

**Keywords:** miR-185, tumor suppressor, oncomiR, oral cancer, gastrointestinal cancer, epigenetics, long non-coding RNA

## Abstract

Digestive tract cancers represent a serious public health issue. In recent years, evidence has accumulated that microRNA miR-185 is implicated in the pathogenesis of this group of highly malignant tumors. Its expression variations correlate with clinical features, such as tumor size, lymph node metastasis, tumor node metastatic stage, survival, recurrence and response to adjuvant therapy, and have diagnostic and prognostic potential. In this review, we compile, evaluate and discuss the current knowledge about the roles of miR-185 in digestive tract cancers. Interestingly, miR-185 is apparently involved in regulating both tumor suppressive and oncogenic processes. We look at downstream effects as well as upstream regulation. In addition, we discuss the utility of miR-185 for diagnosis and its potential concerning novel therapeutic approaches.

## 1. Introduction

Digestive tract cancers are considered a major global health problem. They account for about one-third of global cancer cases and cancer-related mortality [1,2]. Overall, they are responsible for about 4.1 million new cases worldwide and lead to about 3 million deaths each year. Despite advances in treatment strategies, many patients still face poor outcomes; the five-year survival rate of gastric cancer (GC) patients, for example, is less than 10%; the mortality of colorectal cancer (CRC) ranks second among malignant tumors [3] but will be replaced by pancreatic cancer at around 2030 [4]. Digestive tract cancer carcinogenesis is associated with genetic and epigenetic changes [5]. Chromosomal instability—originating from chromosome segregation dysfunction, defective DNA damage response, or induced by *Helicobacter pylori*—as well as microsatellite instability resulting from mismatch repair deficiency are among the most common genetic alterations observed. With respect to epigenetic alterations, aberrant DNA methylation, histone modifications, and altered microRNA (miR) expression are the most important factors involved in pathogenesis [6,7].

Over the years, we have studied the miR-content in cancer tissues and blood samples for both understanding tumor biology and determining the diagnostic potential [8,9,10,11]. miRs are non-protein-encoding RNA molecules that play a critical role in the post-transcriptional regulation of gene expression. The biogenesis of miRs is a multistep process that takes place in the cells’ nucleus and cytoplasm. They are transcribed from intergenic or intronic regions by RNA polymerase II as a primary miRNA and then processed by RNase III enzyme Drosha into stem and loop structures called pre-miRNAs. They are transferred to the cytoplasm and converted to mature double-stranded molecules by the RNase III enzyme Dicer [12,13]. Together with Argonaute proteins, miRs participate in the formation of an RNA-induced silencing complex (RISC), which leads to the degradation or translational suppression of specific target mRNAs. Alteration of miR expression contributes to the development of many diseases. Not surprisingly, miRs are involved in regulating fundamental functions related to cancer, such as cellular proliferation and apoptosis, cell cycle and metabolism, and differentiation [14]. Depending on the physiological environment and pathological condition, miRs may act as oncogenes that induce tumorigenesis or tumor suppressors that restrain tumor development. They also allow a prognosis for different cancer types and have a significant diagnostic and therapeutic potential [15].

**Figure 1:**
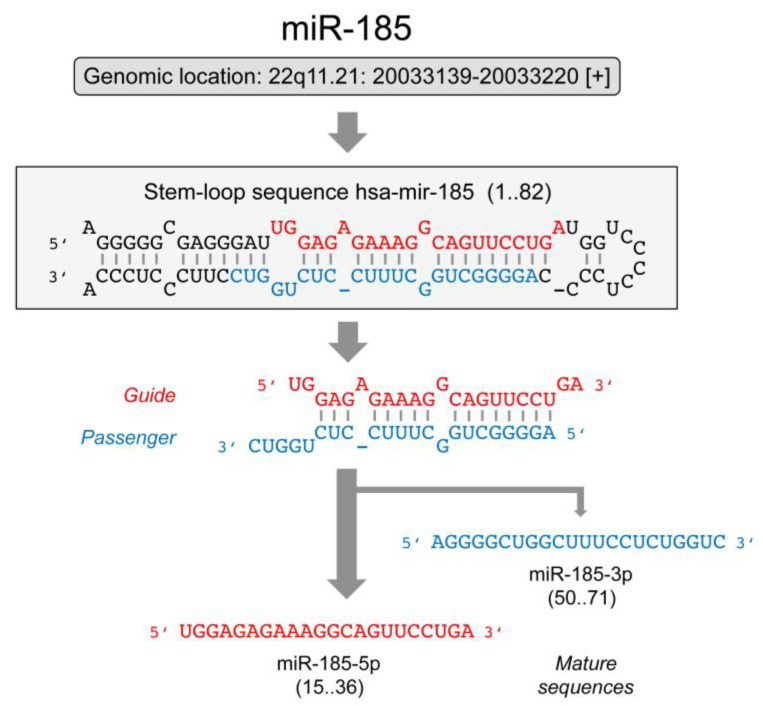
Schematic representation of miR-185 maturation. The gene is located on chromosome 22 (GRC genome assembly number: GRCh38.p14; gene ID: 406961). The stem-loop pre-miRNA, the duplex and the mature sequences are shown. Mostly, the red-labeled strand acts as guide. However, this does change in some instances. In consequence, two mature sequences exist. Sequences were downloaded from miRBase (www.mirbase.com; accessed on 21 September 2022) and the NCBI Gene Database (www.ncbi.nlm.nih.gov; accessed on 21 September 2022).

MicroRNA miR-185 has been found frequently to vary in abundance in samples from cancer patients compared to samples from healthy donors. The gene is located at chromosome 22q11.21. The miR precursor consists of 82 nucleotides and is the source of the two mature molecules miR-185-5p and miR-185-3p; according to available data, miR-185-5p is the predominantly produced molecule (Figure 1).

Dysregulation of miR-185 has been found in various pathological states, and there is much evidence for the misregulation of miR-185 in human cancers. The majority of experimental data indicates that miR-185 exhibits tumor-suppressive activities by affecting many critical biological processes such as cell cycle, epithelial-to-mesenchymal transition (EMT), apoptosis, autophagy, invasion and metastasis. However, fewer but nevertheless several studies have reported that miR-185 acts as an oncogene.

**Figure 2:**
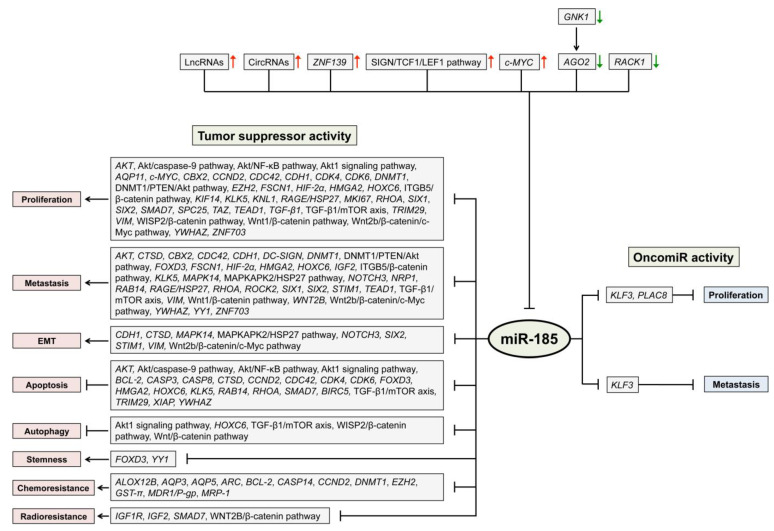
Roles of miR-185 as a tumor suppressor miR or as an oncomiR in digestive tract cancers. Grey boxes show the genes and pathways that are targeted by miR-185. miR-185 has been implicated in the regulation of the tumor-related processes listed on the far left or right. Also, different upstream regulators of miR-185 are presented.

Because of its apparently central role in tumor-related, miR-based regulation and the lack of a compilation and review of the very many different activities related to miR-185 regulation, we set out to summarize the current knowledge about the roles of miR-185 in digestive tract cancers and the molecular mechanisms that are affected. We look at downstream genes regulated by miR-185 and upstream molecules that are involved in controlling miR-185 expression. In addition, we discuss its utility for diagnosis and the potential for therapy. For simplicity, we ordered the review according to the location of the respective tissue in the body, starting with oral cancer and finishing with colorectal tumors. For an overview, a graphical synopsis of the various miR-185 dependent functional consequences is shown (Figure 2). More detailed information is provided as Supplementary Material for particular tumor entities, such as the sample types (tissue, plasma, cell lines, animal tissue) and numbers that were analyzed in the respective study as well as the observed molecular effects. In addition, information is given in the text paragraphs below about which target gene or pathway is known to be affected by miR-185-5p or miR-185-3p, respectively.

## 2. Oral and Pharyngeal Cancers

### 2.1. Biology

There is a growing body of research about the roles of miR-185 in the pathogenesis of oral and pharyngeal cancers (Supplementary Tables S1 and S2). Generally, miR-185 is downregulated in these tumors and acts as a tumor suppressor. Low expression of miR-185-3p in nasopharyngeal carcinoma patients is correlated with poor overall and recurrence-free survival as well as a weak response to radiotherapy by suppressing *SMAD7* and *WNT2B* expression [16,17]. miR-185-3p also inhibits cellular growth and metastasis and promotes apoptosis in nasopharyngeal carcinoma cell lines [17,18]. SMAD7 functions as an antagonist of transforming growth factor β (TGF-β) type I receptor (TGF-βR1) and contributes to the development of various cancers [19]. High miR-185 expression induces apoptosis and autophagy via Homeobox C6 (HOXC6) transcription factor, repressing the oncogenic TGF-β1/mTOR pathway [20]. Low miR-185 expression and high levels of *HOXC6* were found to be associated with lymph node metastasis, higher stages and lower survival. Variations of *HOXC6*, *SMAD7*, *YWHAZ*, *FOXD3, RAB14*, and *ZNF703* exhibited specificity for oral cancers. However, there are no detailed studies about the diagnostic or prognostic value of miR-185 variations.

Enforced expression of miR-185 has reduced dysplasia, cell proliferation and angiogenesis in oral potentially malignant disorders (OPMDs) [21]. There was no significantly different miR-185-5p expression in tonsillar squamous cell carcinoma (TSCC), base of tongue squamous cell carcinoma (BOTSCC) and normal tonsillar tissue. However, high miR-185-5p expression was correlated to human papillomavirus (HPV)-negativity and decreased survival in TSCC/BOTSCC [22]. This implies that miR-185-5p may be more significant for tumor progression than carcinogenesis. In contrast to the above reports, miR-185 up- rather than downregulation in oral squamous cell carcinoma (OSCC) samples was reported by Ramdas et al. [23]. However, this contradictory data is based only on the very low number of just five investigated samples and needs to be considered with much caution.

Besides its involvement in tumorigenesis, the role of miR-185-5p in chemotherapy resistance was investigated. Its upregulation reduced cell viability in cisplatin-resistant tongue and larynx squamous cell carcinoma cell lines by downregulating the genes for aquaporin-3 (*AQP3*), caspase-14 (*CASP-14*) and arachidonate 12R-lipoxygenase (*ALOX12B*) [24]. More recently, miR-185 has been reported to considerably weaken OPMDs by reducing inflammation and inducing apoptosis through Akt/NF-kB and Akt/CASP-9 related pathways. In addition, while no significant difference in the miR-185-5p serum levels was found in oropharyngeal cancer patients and healthy subjects, serum levels were increased following radiotherapy and allowed an accurate prediction of severe radiation-induced xerostomia [25].

### 2.2. Regulation

**Figure 3:**
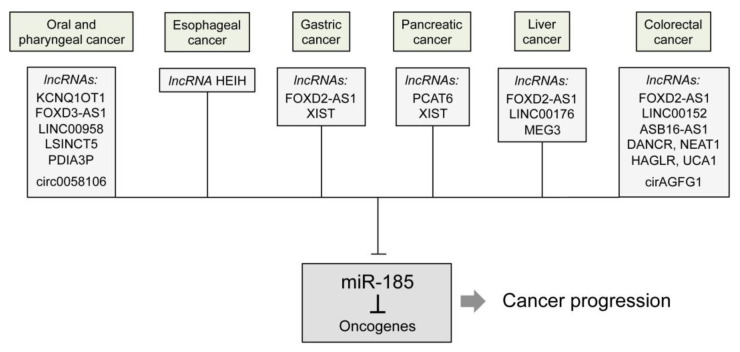
LncRNAs and circRNAs are shown which act as major upstream regulators of miR-185 in digestive tract cancers and drive cancer progression by suppressing miR-185.

Long non-coding RNAs (lncRNAs) and circular RNAs (circRNAs) are major upstream regulators of miR-185 (Figure 3). Higher levels of lncRNA Forkhead box D3 antisense RNA1 (FOXD3-AS1) led to increased expression of *FOXD3* and the development of nasopharyngeal carcinoma through absorption of miR-185-3p [26]. Conversely, overexpression of miR-185-3p in nasopharyngeal carcinoma cells reversed oncogenic activities of FOXD3-AS1. circRNA circ0058106 suppressed miR-185-3p in hypopharyngeal squamous cell carcinoma thereby increasing proliferation, metastasis and EMT by activating the Wnt2b/β-catenin/c-Myc pathway [27]. Third, lncRNA LINC00958 promoted OSCC through sponging miR-185-5p [28]. Its overexpression directly suppressed miR-185-5p and indirectly enhanced *YWHAZ* expression, which is known to function as an oncogene [29]. Forced expression of miR-185-5p, however, counteracted this effect and suppressed *YWHAZ* both at mRNA and protein levels. lncRNA PDIA3P promoted OSCC progression through sponging miR-185-5p, thereafter activating *CCND2* (cyclin D2). Interestingly, the *CCND2* mRNA level was significantly higher in OSCC tissues, but its protein level was downregulated by miR-185-5p overexpression [30]. As a fifth molecules, lncRNA LSINCT5 was aberrantly upregulated in OSCC and drove tumor progression by decreasing miR-185-5p and increasing *ZNF703* levels [31]; *ZNF703* has been characterized to be an oncogene in OSCC and a target of miR-185-5p. Finally, lncRNA KCNQ1OT1 accelerated OSCC tumorigenesis and reduced apoptosis by direct inhibition of miR-185-5p [32].

## 3. Esophageal Cancer

### 3.1. Biology

There are inconsistent reports about the function of miR-185 in esophageal cancers (Supplementary Table S3). Some evidence shows that miR-185 is upregulated in esophageal squamous cell carcinoma (ESCC) patients and associated with tumor node metastasis (TNM) stages [33,34,35]; this may have diagnostic potential. On the other hand, high expression of miR-185 led to a favorable prognosis in esophageal carcinoma patients and suppressed the malignancy and growth of esophageal carcinoma cells in vivo [36]. Also, upregulation of miR-185 in ESCC cells reduced proliferation, invasion, migration and metastasis through downregulation of the genes of the receptor for advanced-glycation end products (*RAGE*) and transcription factor *Six1* [37,38]. RAGE activates several signaling pathways in various cancers and is involved in processes, such as cell proliferation, autophagy and apoptosis [39]. The homeobox protein Six1 plays an oncogenic role in various cancers [40]. miR-185 has also been implicated in the radioresistance of esophageal cancer. In two studies, Su et al. [41] and Zheng et al. [42] produced miR profiles of radioresistant esophageal cancer cells and reported downregulation of miR-185 expression compared to the radiosensitive parental cell line. High plasma levels of miR-185 were also observed in ESCC patients who responded to radiotherapy [43].

### 3.2. Regulation

lncRNA that is highly expressed in hepatocellular carcinoma exhibits oncogenic activities in esophageal carcinoma by upregulating the kallikrein-related peptidase 5 (*KLK5*) gene through sponging miR-185 [36]. Liu and colleagues [44] observed that the lncRNA KLF3-AS1, which exhibits a low expression in tumor tissues, functions as a competing endogenous RNA (ceRNA) for miR-185-5p in ESCC. The Krüppel-like factor 3 (*KLF3*) gene exhibits a variety of biological effects, such as regulation of differentiation and apoptosis. The results suggest that *KLF3* acts as a tumor suppressor in ESCC by inhibiting migration and invasion of tumor cells. Loss of KLF3-AS1 results in high levels of miR-185-5p, which in turn lead to *KLF3* downregulation (Figure 3).

## 4. Gastric Cancer

### 4.1. Biology

Generally, miR-185-5p is downregulated in gastric cancer (GC) promoting cell proliferation and invasion while simultaneously inhibiting apoptosis and necrosis [45] (Supplementary Table S4). Several studies suggest a miR-185-dependent mechanism for regulating the antitumor effects of *GKN1*. Gastrokine 1 (GKN1) is a protein expressed in gastric mucosa and exhibits gastroprotective effects [46,47]. The expression levels of *GNK1* and miR-185 are low in GC patients and negatively correlated with the methylation status of the CpG island methylator phenotype and high transcript levels of both the DNA methyltransferase 1 (*DNMT1*) and the enhancer of zeste homolog 2 (*EZH2*) gene [48]. miR-185 is induced by GKN1 and required for GKN1’s tumor-suppressive activities [49]. Interestingly, GKN1 increased the expression of miR-185 in GC cell lines by suppressing c-Myc, which was recognized as a suppressor of miR-185 by binding to its promoter. Increased miR-185 expression in GC cell lines led to cell cycle arrest and reduced proliferation by inactivating *DNMT1* and *EZH2* and increasing the expression of the cyclin dependent kinase inhibitor 2A (*CDKN2A*) and E-cadherin (*CDH1*) genes. The increased expression was the result of GKN1/miR-185/DNMT1/EZH2–mediated hypomethylation of the promoter regions. The N-terminal hydrophobic region and BRICHOS domain of GKN1 were sufficient for its tumor-suppressive functions [50]. Downregulated *GKN1* and miR-185 have also been shown to be negatively correlated with the high expression of ras homolog gene family member A (*RHOA*) [51]. GKN1 promotes the miR-185 expression level in GC cell lines and significantly inhibits cell migration and invasion by repressing *RHOA* in a miR-185 dependent manner. RhoA is a member of the Rho subgroup of the Ras superfamily and exhibits oncogenic activities [52].

Some studies have reported that miR-185 is involved in regulating the chemosensitivity of GC. miR-185 is downregulated in drug-resistant GC cells. miR-185 inhibition increased resistance to chemotherapeutic drugs by upregulating multidrug resistance (MDR) genes [53]. Inversely, enhanced expression led to more chemosensitivity of GC cells by reducing the expression of apoptosis repressor with caspase recruitment domain (*ARC*) both in in vitro and in vivo [54]. An upregulation of miR-185 upon cisplatin or doxorubicin therapy was the result of direct binding of Runt-related transcription factor 3 (RUNX3) to the binding site 3 (BS3) region of the miR-185 promoter, which finally triggered the promoter activity of miR-185. Tan et al. [55] reported the involvement of miR-185 in zinc finger protein 139 (*ZNF139*) induced enhancement of MDR in GC cells. ZNF139 was confirmed to be an upstream regulator of miR-185 that diminishes miR-185 transcription. This increased MDR characteristics in GC cells through the induction of the MDR-associated genes *MDR1/P-gp*, *MRP* and *BCL-2*. In another study, a significantly lower level of serum miR-185 was observed in patients with advanced GC, but it increased after neoadjuvant chemotherapy with the SOX regimen [56]. This could be used as a biomarker to predict the outcomes of neoadjuvant chemotherapy.

miR-185 levels correlate with advanced clinical stage, lymph node metastasis and poor clinical outcomes [57]. Additionally, miR-185-5p expression was associated with clinicopathological characteristics, including tumor size, differentiation, and lymphatic metastasis [58]. Enforced expression suppressed the genes *DNMT1* and tripartite motif-containing 29 (*TRIM29*) and resulted in a rescue of GC proliferation and metastasis, as well as an induction of apoptosis and cell cycle arrest [59]. TRIM29 is a transcription factor that has shown oncogenic or tumor-suppressive activities dependent on the tumor type. One of the mechanisms of the tumor suppressive activity of miR-185 is related to its ability to directly suppress the TRIM29-Wnt/β-catenin signaling axis. Interestingly, miR-185-5p mimics significantly increased apoptosis of GC cells by decreasing expression of *BCL-2* and the gene of the X-linked inhibitor of apoptosis protein (*XIAP*), simultaneously increasing CASP-8 and CASP-3 expression and activities [58]. Enforced overexpression of miR-185-3p in GC cells led to a strong inhibition of EMT and migration and increased apoptosis through inactivation of the PI3K/Akt axis by downregulation of the cathepsin D gene (*CTSD*) [60].

Conversely to the data above, some studies reported high miR-185 levels in GC (Supplementary Table S4). Yao et al. [61], Treece et al. [62], and Zhang et al. [63] found considerable upregulation of miR-185 in GC patients, for example. Looking for biomarkers for GC detection, Zhou et al. [64] investigated the levels of miRs in plasma, GC tissues and plasma exosomes derived from GC patients. They observed an increased level of miR-185 in all three sample types. In addition, a higher level was detected in the plasma of patients with high- as compared to low-stage GC. In a similar study, miR-185-5p expression was found to be high in serum, tissue, and serum exosomes and, along with other miRs, showed diagnostic value for GC detection [65]. High miR-185 levels have also been associated with chemoresistance [66].

### 4.2. Regulation

In addition to lncRNAs, the genes *GNK1*, *c-Myc*, *ZNF139*, and *RUNX3* encode specific upstream regulators of miR-185 in GC. Interestingly, other lncRNAs were identified to regulate the expression of miR-185 in GC than in oral, pharyngeal or esophageal cancers (Figure 3). lncRNA XIST has been shown to regulate miR-185 expression [67] and functioned as a ceRNA for miR-185 to decrease miR-185-induced TGF-β1 suppression and accelerate GC progression. Wu and colleagues [68] reported that lncRNA FOXD2-AS1 was able to increase apatinib resistance by direct suppression of miR-185-5p and decreased the inhibitory effects of miR-185-5p on *CCND2*. By contrast, ectopic expression of miR-185-5p could suppress the FOXD2-AS1 effect on apatinib resistance and sensitize GC cells to apatinib through *CCND2* suppression [68].

## 5. Liver Cancer

### 5.1. Biology

There is growing evidence that miR-185 expression in the liver has implications through regulating several oncogenes in hepatocellular carcinoma (HCC) (Supplementary Table S5). This includes the gene cell division cycle 42 (*CDC42*) which belongs to the Rho GTPase family; its oncogenic activities are well documented in various cancers [69]. The Rho-associated coiled-coil containing protein kinase 2 gene (*ROCK2*) encodes for a serine/tyrosine kinase that is involved in controlling HCC proliferation, metastasis and chemoresistance [70]. *CDC42* and *ROCK2* are direct targets of miR-185 and overexpressed in HCC cells due to miR-185 deficiency [71,72]. When miR-185-5p expression was restored, cell migration and invasion capacity were dramatically reduced. A low level of miR-185-5p expression in HCC tissues was also positively correlated with aggressive clinicopathological features, such as high TNM stage and lymph node metastasis.

Integrin-β5 (ITGB5) is involved in the cell interaction with the extracellular matrix (ECM) and promotes HCC carcinogenesis by activation of the WNT/β-catenin pathway; homeobox gene *SIX2* is a known oncogene. Both are targeted by miR-185 [73,74]. Through the inactivation of the WNT/β-catenin pathway in an ITGB5-dependent manner and the suppression of *SIX2*, miR-185 exerts anti-growth and anti-metastatic effects on HCC cells and reverses EMT. Qadir et al. [75] observed a low miR-185 expression in HCC tissues infected with hepatitis B (HBV) and hepatitis C viruses (HCV) as well as three different HCC cell lines. This study indicated that restoration of miR-185 expression suppressed *DNMT1* expression and *PTEN* promoter methylation, increased *PTEN* expression and consequently *PTEN*-induced Akt inhibition and thus prevented HCC growth. Epigenetic control of *PTEN* by miR-185 has also been shown in cholangiocarcinoma [76]. Additionally, miR-185 was found to inhibit proliferation and cell cycle progression and induce apoptosis and autophagy in HCC cells by targeting different genes in the Akt signaling pathway, including *AKT1*, *RICTOR* and *RHEB* [77].

Downregulation of miR-185 is considered a prognostic marker of HCC. Zhi and colleagues [78] reported accurate prognosis of survival and tumor recurrence in patients with early-stage HCC, a finding that was confirmed by others [79]. Furthermore, miR-185 mimics caused a considerable suppression of cell growth and invasion in HCC cell lines. Serum miR profiling in HCV-positive HCC patients recognized six dysregulated miRs, which showed a high diagnostic accuracy for discriminating HCV-positive HCC from non-HCC individuals [80]. miR-185-5p was one of them, exhibiting lower abundance in cancer patients.

As opposed to the above-mentioned publications, a few studies have reported upregulation of miR-185 and an oncogenic activity in HCC (Supplementary Table S5). Wen and colleagues [81] evaluated the miR profiles of plasma derived from HBV-positive HCC patients. They highlighted eight overexpressed miRs, including miR-185-5p, with diagnostic potential. Likewise, Tang and colleagues [82] determined miR profiles in HCC clinical samples and found 20 miRs that were related to venous metastasis, again including miR-185. Placenta-specific 8 (PLAC8) or Onzin is a highly conserved cysteine-rich protein that is downregulated in HCC and acts as a tumor suppressor. High expression of miR-185-5p actually downregulates *PLAC8* expression [83].

### 5.2. Regulation

Some lncRNAs have been shown to be involved in miR-185 regulation (Figure 3). One molecule is lncRNA FOXD2-AS1. Its knockdown effectively increased miR-185 expression, thus suppressing *AKT* and subsequently reducing proliferation, invasion and migration [84]. Linc00176 also seems to play an oncogenic role. Upon its knockdown, there is a release of miR-185-5p and an induction of cell cycle arrest and necroptosis [85]. LncRNA MEG3 is downregulated in HCC due to methylation of its promoter region. It exerts tumor-suppressive activities via regulation of p53 expression [86]. Zamani et al. [87] reported that dendrosomal curcumin increases miR-185 expression in HCC cells and thereby induces promoter DNA hypomethylation and upregulation of *MEG3* through *DNMT1* targeting.

Besides lncRNA molecules, miR-185 is regulated by a different mechanism via the receptor for activated protein kinase C (RACK1) [88]. RACK1 is downregulated in HCC tissues. While it is not needed for maturation, it is required for full miR-185 functioning. RACK1 interacts with a part of the Dicer complex called KH-type splicing regulatory protein (KSRP) and is necessary for the recruitment of mature miR-185 to RISC. Knockdown of *RACK1* caused a weaker miR-185-mediated inhibition of the expression of target genes in HCC cells. Another process involves ELK1. It is a transcription factor that belongs to the ternary complex factor subfamily of the ETS family and is involved in viral life cycles and virus-associated diseases. Fan et al. [89] found that miR-185-5p inhibits HBV replication and gene expression in HCC cell lines in an ELK1-dependent manner and also reduced HBV preS1 promoter activity by inhibiting *ELK1*. A rescued *ELK1* expression reversed the suppressive effects of miR-185-5p.

## 6. Pancreatic Cancer

### Biology and Regulation

Pancreatic cancer (PDAC) mortality is close to incidence and will become the second most frequent cause of cancer-related death by about 2030. Concerning miR-185 (Supplementary Table S6), an inverse association between lncRNA XIST and miR-185-5p has been described. Enforced expression of miR-185-5p or knockdown of XIST in PDAC cell lines reduced cell proliferation and triggered cell cycle arrest and apoptosis. XIST also suppressed miR-185-5p through direct interaction, which in turn inhibited oncogene *CCND2* [90]. lncRNA PCAT6 promotes progression and tumorigenesis of PDAC through sponging of miR-185-5p and a resulting upregulation of the chromobox 2 (*CBX2*) gene [91]. In contrast, miR-185-5p mimics interfered with oncogenic activities of PCAT6. Overexpression and oncogenic activities of CBX2 have also been reported in several other cancers. In another study, increased abundance of transcriptional coactivator with PDZ-binding motif (TAZ) has been shown in tissues, serum and pancreatic fluid of PDAC patients and is negatively correlated with miR-185 expression. TAZ is a 14-3-3 binding protein with confirmed oncogenic activities in PDAC [92]. miR-185 inhibited the proliferation of PDAC cells by downregulating the TAZ-encoding gene *TAFAZZIN* through the direct targeting of the 3′-UTR of its mRNA [93]. Rather than suppressing tumor-related activities, Gao et al. [94] reported that upregulation of miR-185 contributes to PDAC development by downregulation of *NTRK3* and *CORO2B*. The analysis was based on data obtained from only few tissues, however. NTRK3 belongs to the neurotrophin receptor family and modulates cell survival, while CORO2B is an actin-binding protein. Diagnostically, higher levels of miR-185 and six other miRs (miR-20a, miR-21, miR-24, miR-25, miR-99a, and miR-191) were found in a comparison of PDAC patients to healthy donors and patients with chronic pancreatitis [95]. The panel could discriminate different tumor stages with high sensitivity and specificity. Overall, however, miR-185 has not been found to contribute significantly to blood-based diagnosis of PDAC [96].

## 7. Colorectal Cancer

### 7.1. Biology

Colorectal cancer (CRC) is the third most common cancer in the world, with a high mortality rate [97]. The role of miR-185 in CRC has been widely evaluated. Most studies confirm that miR-185 is downregulated and exhibits tumor suppressing activity via several cellular processes (Supplementary Table S7). The Wnt/β-catenin pathway is one of the targets implicated in CRC development. miR-185 upregulation reduced aggressive features of CRC by directly targeting *WNT1* as well as *MYC* and *CCND1* further downstream [98,99]. Urothelial carcinoma-associated 1 (UCA1) is an lncRNA that is significantly upregulated in CRC and involved in its progression [100]. UCA1 functions as a sponge for miR-185-5p, thereby activating the WNT1-inducible signaling pathway protein 2 (WISP2)/β-catenin pathway and promoting CRC.

Other studies have shown that miR-185-5p and miR-185-3p considerably reduced CRC growth and its metastatic and angiogenic potential by directly suppressing *RHOA* [101], *CDC42* [101], *c-MYC* [102], and the gene of aquaporin 5 (*AQP5*) [103]. The stromal interaction molecule 1 (*STIM1*) gene is also directly affected [104]; it is an endoplasmic reticulum calcium sensor that activates the store-operated calcium influx when ER discharges calcium. Further, insulin-like growth factor 1 receptor (*IGF1R*) and insulin-like growth factor 2 (*IGF2*) are targeted by miR-185 [105]. IGF1R is a tyrosine kinase receptor that plays a pivotal role in angiogenesis, growth, metastasis and resistance to apoptosis in CRC. IGF2 triggers the signaling pathway associated with proliferation and survival of CRC through IGF1R. Hypoxia-inducible factor-2α (*HIF-2α*) is another verified target of miR-185 [106]. It increases hypoxic tumor cell proliferation through *c-Myc* activation and leads to CRC progression by deregulating iron homeostasis. Additionally, suppression of these genes by miR-185 noticeably inhibited EMT, increased apoptosis and reduced CRC resistance to chemotherapy and radiotherapy [103,104,105].

Yuan and colleagues [107] reported that miR-185 blocks *MMP-9* and *VEGF* expression as well as the metastatic ability of CRC cells by repressing *DC-SIGN*. DC-SIGN is a C-type lectins domain family 4 member and abundantly expressed in immature dendritic cells. The miR-185 repression of *DC-SIGN* prevents β-catenin translocation to the nucleus of CRC cells by inactivation of the PI3K/Akt/GSK-3β pathway and the DC-SIGN/TCF1/LEF1 pathway could directly downregulate miR-185 in CRC. Li et al. [108] found a low-frequency 3′-UTR variant rs12915554 in *GREM1*—a member of the TGF-β superfamily—associated with enhanced CRC susceptibility. Interestingly, this variation stabilized the *GREM1* transcript by disturbing the binding of miR-185-3p in CRC cells. In another paper, it was reported that miR-185-3p is downregulated in CRC due to the impairment of argonaute 2 (AGO2), a key regulator in miR processing [109]. Treatment with a miR-185-3p mimic could reduce metastasis by downregulating *NRP1*. NRP1 has been shown to interact with different ligands and receptors to increase tumorigenesis and EMT [110].

miR-185 expression has shown potential in diagnosis and prognosis of CRC. Significantly lower miR-185 expression was found in cancerous than non-cancerous tissues [111,112]. In addition, this was associated with advanced clinical stage and metastasis. Downregulation was also reported in CRC patients with subsequent relapse. miR-185 had a prognostic value for metastasis-free survival in combination with four other miRs [113]. Similarly, the miR-185 abundance in exosomes derived from serum was reduced in samples from patients with recurrence of liver metastasis [114]. The expression of miR-185 in circulating tumor cells derived from metastatic CRC patients during a chemotherapy course was transient and fluctuating [115]. Low miR-185-5p expression in CRC tissue samples is also associated with liver metastasis [116], while higher plasma levels were predictive for chemotherapy response. Expressions of miR-185 and that of affected genes, such as glutathione peroxidases 2 (*GPX2*) and selenophosphate synthetase 2 (*SEPHS2*), decreased considerably in tumor cells grown in selenium-deficient medium [117].

In contrast to the majority of findings, there are few studies that reported miR-185 upregulation in CRC, actually promoting carcinogenesis (Supplementary Table S7). Akcakaya et al. [118] showed correlation with poor survival and development of metastatic disease in CRC patients, implying that miR-185 might have a negative prognostic role in CRC. Recently, another study identified that miR-185-5p-induced suppression of AT-rich interaction domain 1A (*ARID1A*), a tumor suppressor gene, was associated with poor prognosis and adverse outcomes [119]. Zhang et al. [120], finally, reported that miR-185 was expressed at a high level in colon cancer stem cells and seems to be important in maintaining stemness. Moreover, its expression was significantly increased during the transformation of normal intestine treated with a carcinogenic agent [121].

### 7.2. Regulation

As for other types of digestive tract cancers, lncRNAs have been identified to trigger CRC progression via miR-185 regulation (Figure 3). High expression of lncRNA UCA1 in CRC cells led to a notable increase in the migration, invasion and EMT by binding to miR-185-5p, which directly activated the MAPK14/MAPKAPK2/Hsp27 axis [122] and repressed *NOTCH3* [123]. In another study, overexpressed LINC00152 promoted CRC growth by sponging miR-185-3p and upregulating its direct target fascin actin-bundling protein 1 (*FSCN1*). However, miR-185-3p activation partially reversed this effect [124]. NEAT1 increased CRC metastasis through miR-185-5p absorption and *IGF2* induction. By contrast, miR-185-5p mimics decreased NEAT1-induced CRC metastasis [125]. As shown also in HCC, FOXD2-AS1 downregulated miR-185-5p and thus upregulated its target *CDC42*. This resulted in an increased proliferation, migration and invasion capacity of CRC cells [126]. In turn, miR-185-5p overexpression considerably reversed the aggressive features. HAGLR is a recently discovered lncRNA that is aberrantly expressed in many malignancies. Recently, it was reported that HAGLR is upregulated in CRC and promotes cancer in a xenograft model by targeting miR-185-5p, leading to upregulated *CDK4* and *CDK6* [127]. This effect could be reversed by overexpressing miR-185-5p. Another study showed that high expression of circRNA ArfGAP with FG repeats 1 (circAGFG1) increased the expression of transcription factor YY1, induced *CTNNB1* (β-catenin) and triggered metastasis and stemness by adsorbing miR-185-5p [128]. LncRNA differentiation antagonizing non-coding RNA (DANCR) has been shown to be upregulated in CRC [129]. Overexpression increases high mobility group A2 (*HMGA2*) expression by repressing miR-185-5p and promotes CRC progression. ASB16 antisense RNA 1 (ASB16-AS1) has also been shown to drive CRC progression by downregulating miR-185-5p and upregulating TEA domain transcription factor1 (*TEAD1*) as the main effector of Hippo signaling [130].

## 8. Conclusions

Dysregulation of miR-185 is strongly implicated in the pathogenesis of digestive tract cancers and associated with clinical outcome. The fact that the molecule is relevant to all cancers indicates that it has a central role in regulating tumor biology. Both miR-185-5p and miR-185-3p are functionally active in the various tumors and numerous target genes or pathways have been reported (Table 1). On the basis of the currently available data, it seems as if more genes are affected by miR-185-5p than miR-185-3p. However, this could be accidental or due to a bias in the studies performed. In addition, quite a few analyses did not specify the variant and only reported a role of miR-185 globally. It is reassuring, however, that no gene was found to be regulated by both miR-185-5p and miR-185-3p in different tumor entities. For three genes—*CDC42*, *PLAC8*, and *RHOA*—for which no information about the variant is available in some tumor types, comparison between different datasets or tumor entities may suggest which miR-185 version is responsible for their regulation. For these and all others, however, comparison to the respective target mRNA would provide the relevant information.

The fact that most regulation seemingly occurs via miR-185-5p and less via miR-185-3p suggests that oncogenic and tumor suppressive functions could be linked to one of the two mature miRs, respectively. Alternatively, the use of either miR-185 variant could be organ-related. However, no such correlation was apparent from the available data.

While many functional aspects of miR-185 variation have been demonstrated, the reported analyses have been focusing on particular gene functions, providing only many fragmented views while lacking a more comprehensive understanding of parallel or synergistic regulative processes and their interaction. A good example for this is the reports about the regulation of miR-185 in oral cancer and its functional consequences (Table 2). Each study looked at only one regulator of miR-185 and the effects that the reduced miR-185 level had for one or a few genes. While the same process—reduction of miR-185—was described in all cases, there was basically no apparent overlap between the results of the six studies.

About three quarters of the publications discussed in this review have reported that miR-185 predominantly exhibits tumor-suppressive functions. Conversely, however, some studies suggest that it functions as an oncomiR. Such contradictory results were reported for nearly all mentioned tumor forms. From the publications and data looked at as part of this review, there was no factor with apparent responsibility for this striking difference. However, the number of publications indicating an oncomiR function is too large to merely assume erroneous studies. In several cases, the relevant target genes responsible for the actual oncogenic activity have not been described. Further analyses are required to elucidate the reasons for the differences.

To some extent, the contradictory results might be associated with differences in methodologies and analyzed material. For example, Cao et al. [122] found that miR-185 was downregulated in CRC cell lines, SW480, SW620, and HT-29, compared to a non-cancerous colon cell line and miR-185 mimics inhibited cell proliferation and migration. Baldi et al. [119] reported instead overexpression of miR-185 in CRC cell lines HCT116 and LoVo and thereby oncogenic activities. Analyzing miR-185 expression in only relatively few samples may also result in contradictory results. Interestingly, however, there are results that were produced with the same methodology and on the same cell lines but nevertheless led to opposing results. Zou et al. [83] found that overexpression of miR-185 triggered oncogenic activities and enhanced cell viability in HCC cell lines Huh-7 and HepG2. By contrast, Tran et al. [85] showed that miR-185 inhibition rescued the same two HCC cell lines from cell death. One cannot exclude entirely that an undetected heterogeneity between the actual cell lines used in the different laboratories may be a factor, which would be difficult to unravel retrospectively, though.

In terms of biology, the contradictory roles of miR-185 may be dependent on its expression level and cellular abundance in relation to the expression of its targets (dose-dependent effects), as has been reported for other miRs [131]. Another explanation could be genomic heterogeneity. This is a known characteristic of human cancers and has been observed in tumors derived from different patients and even within a tumor from an individual patient [132]. Due to the context-dependent function of miRs [133], contextualization of miR-185 in specific genetic backgrounds may be an explanation for the functional discrepancies.

Several lncRNAs and circRNAs as well as *AGO2*, *c-Myc*, *GNK1, RUNX3,* *RACK1*, *ZNF139*, and the DC-SIGN/TCF1/LEF1 pathway have been reported as upstream regulators implicated in controlling miR-185 abundance and function in digestive tract cancers. Of these, lncRNA FOXD2-AS1 is an lncRNA that commonly regulates miR-185 abundance in colorectal, gastric, and liver cancers. Additionally, lncRNA XIST regulates miR-185 in both gastric and pancreatic cancers. However, identification of these overlapping processes in regulation is rather coincidental. Still missing to date are comprehensive studies of the mechanisms involved in the control and regulative processes of miR-185; synergies or complementation may have an effect that can only be revealed by such analyses. Moreover, epigenetic mechanisms, such as histone modifications and promoter DNA methylation, have not been investigated much.

In terms of functioning as a biomarker, several studies looked at the diagnostic and prognostic potential of miR-185 in tissue, plasma/serum or exosomes derived from patient blood. miR-185 is likely to be informative in combination with other features, acting more as a pan-cancer marker. However, there is still too little knowledge about the rules governing the changes and the processes that are possibly involved, such as an exosomal transfer of miR-185. Furthermore, the association between miR-185 expression and tumor microenvironment and immune cell infiltrations has not been examined yet. Particularly for CRC, miR-185 could even hold some promise as a therapeutic target, but much more data and detailed information will be required to assess its real potential.

## Figures and Tables

**Table 1:** Target genes of miR-185. Genes or pathways affected by miR-185-5p are labeled in **red**, the ones targeted by miR-185-3p in **blue**. Genes or pathways for which this information is not available from the literature are indicated in black letters.

Cancer Tissue	Identified by In Vitro Experiments	Predicted from Data	Verified by In Vivo Experiments
**Mouth and pharynx**	*ALOX12B*, *AQP3,CASP-14,CCND2*, *FOXD3*, *HOXC6*, *RAB14,SMAD7*, TGF-β1/mTOR axis, *WNT2B*, *YWHAZ*, *ZNF703*, Akt/CASP-9 pathway, Akt/NF-κB pathway, Wnt2b/β-catenin/c-Myc pathway		*CCND2*, *FOXD3*, *HOXC6*, *YWHAZ*, *ZNF703*, Akt/CASP-9 pathway, Akt/NF-κB pathway
**Esophagus**	*KLF3*, *KLK5,RAGE*, *SIX1*		*KLK5,* *RAGE*
**Stomach**	*ARC*, *BCL-2*, *CASP-3*, *CASP-8*, *CCND2*, *CTSD,DNMT1*, *EZH2,GST-π*, *MDR1/P-gp*, *MRP-1,RHOA*, *TGF-β1*, *TRIM29*, *XIAP*, Wnt/β-catenin pathway	*AQP5*, *ESRRA*, *RAC3*, *RGS14, ZNFN1A4*	*ARC,CTSD, DNMT1*, *EZH2*, *RHOA*
**Liver**	*AQP11, AKT, CDC42*, *CDH1, DNMT1*, *DNMT3A*, *DNMT3B*, *ELK1*, *KIF14,KNL1, MEG3,MKI67, PLAC8,ROCK2*, *SIX2*, *SPC25, VIM,* Akt1 signaling pathway, DNMT1/PTEN/Akt pathway, ITGB5/β-catenin- and PLAC8/Wnt/β-catenin pathway		*CDC42*, DNMT1/PTEN/Akt pathway, ITGB5/β-catenin pathway
**Pancreas**	*CBX2*, *CCND2*, *TAFAZZIN*	*CORO2B, NTRK3*	*CBX2*, *CCND2*, *TAFAZZIN*
**Colon or** **rectum**	*AQP5*, *ARID1A,c-MYC*, *CDC42*, *CDK4*, *CDK6*, *DC-SIGN*, *FSCN1*, *GREM1*, *HIF-2α*, *HMGA2*, *IGF1R*, *IGF2*, *MAPK14*, *NOTCH3*, *NRP1*, *RHOA*, *STIM1*, *TEAD1*, *YY1*, MAPKAPK2/HSP27 pathway, WISP2/β-catenin pathway, Wnt/β-catenin pathway		*AQP5*, *CDC42*, *CDK4*, *CDK6*, *DC-SIGN*, *FSCN1*, *HIF-2α*, *HMGA2*, *IGF2*, *MAPK14*, *NRP1*, *STIM1*, *TEAD1,YY1*, MAPKAPK2/HSP27 pathway, WISP2/β-catenin pathway

**Table 2:** Example for the fragmented description of miR-185 regulation and activity in oral cancer.

Upstream Regulator	Process	Affected Gene or Pathway/Function	Ref.
FOXD3-AS1	Reduction of miR-185 level	*FOXD3*	[26]
irc0058106		Wnt2b/β-catenin/c-Myc pathway	[27]
LINC00958		*YWHAZ*	[28]
PDIA3P		*CCND2*	[30]
LSINCT5		*ZNF703*	[31]
KCNQ1OT1		Increased tumorigenesis and reduced apoptosis	[32]

## Supplementary Materials

The following supporting information can be downloaded at: https://www.mdpi.com/article/10.3390/ncrna8050067/s1, Table S1: miR-185 expression in oral cancers; Table S2: miR-185 expression in nasopharyngeal cancers; Table S3: miR-185 expression in esophageal cancers; Table S4: miR-185 expression in gastric cancers; Table S5: miR-185 expression in hepatocellular carcinoma (HCC); Table S6: miR-185 expression in pancreatic cancer (PDAC); Table S7: miR-185 expression in colorectal cancer (CRC).
