# Supplementary material for: The Roles of microRNA miR-185 in Digestive Tract Cancers
Source: Noncoding RNA. 2022 Oct 8;8(5):67. doi: 10.3390/ncrna8050067 (PMC9609348; doi:10.3390/ncrna8050067)
Supplement: Supplementary file 1 [file ncrna-08-00067-s001.zip › ncrna-1904897-supplementary.pdf]

Supplementary Table S1. miR-185 expression in oral cancers.

Cancer type	miR-185 expression	Affected gene / pathway	Functional consequences	Samples analyzed	Ref.
OPMDs	Down	Akt/NF-κB pathway, Akt/CASP-9 pathway	Decreased inflammation, apoptosis, cell proliferation, and angiogenesis	- Cell line Cal27<br>- Animal tissue	[21]
OSCC	Down	*AQP3, CASP-14, ALOX12B*	Increased sensitivity to cisplatin	- Cell lines SCC-11, SCC-11M, SCC-25, and SCC-25CP	[24]
OSCC	Down	*CCND2*	Decreased *CCND2* expression and cell viability	- Tissue (n=58)<br>- Cell lines SCC4, SCC9, SCC1, SCC25, TU183, HSU3, FADU, OEC-M1, SNU1041, and SCC15	[30]
OSCC	Down	*RAB14*	Reduced *Rab14* expression and invasion, increased apoptosis	- Tissue (n=60)<br>- Cell lines Tca-8113, SCC15, Cal-27, HSC-3, and SCC-090	[32]
OSCC	Down	*YWHAZ*	Upregulation decreases *YWHAZ* and tumorigenesis	- Tissue (n=30)<br>- Cell lines SCC9, SCC25, CAL27, and SCC15	[28]
OSCC	Down	*ZNF703*	Promotion of proliferation and migration	- Tissue (n=23)<br>- Cell lines Fadu, SCC-25, CAL-27, OMEC, and Tca8113	[31]
Hypopharyngeal squamous cell carcinoma	Down	Wnt2b/β-catenin/c-Myc pathway	Increased proliferation, metastasis and EMT	- Cell lines Fadu, TU686, TU212, and Detroit562<br>- Animal tissue	[27]
TSCC/BOTSCC	-	-	Differentiation of HPV^+ and HPV^- TSCC/BOTSCC, low expression related to increased survival in all patients, associated with decreased survival in HPV^- TSCC/BOTSCC	- Tissue (n=168)	[22]

OPMD, oral potentially malignant disorder; OSCC, oral squamous cell carcinoma; TSCC, tonsillar squamous cell carcinoma; BOTSCC, base of tongue squamous cell carcinoma

Supplementary Table S2. miR-185 expression in nasopharyngeal cancers.

miR-185 expression	Affected gene / pathway	Functional consequences	Samples analyzed	Ref.
Down	*FOXD3*	Upregulation diminishes cell stemness, invasion, migration, and viability and enhances apoptosis	- Tissue (n=52)<br>- Cell lines C666-1 and HK-1<br>- Animal tissue	[26]
Down	*HOXC6*, TGF-β1/mTOR axis	Associated with increased apoptosis and autophagy, reduced proliferation and invasion, clinicopathological features, and a good prognosis	- Tissue (n=126)<br>- Cell lines 5-8F, HNE-1, and HNE-2	[20]
Down	*SMAD7*	Correlated to clinicopathological features, radioresistance, increased apoptosis, decreased cell growth, and a good prognosis	- Tissue (n=80)<br>- Cell lines CNE-2 and HEK-293T	[18]
Down	WNT2B/β-catenin pathway	Decreased radioresistance	- Tissue (n=15)<br>- Cell lines CNE-2, HNE-1, 5-8F, and 6-10B	[16]
Down	*WNT2B*	Decreased invasion and metastasis	- Cell lines CNE-1, CNE-2, HNE-1, and HNE-2, 5-8F	[17]

Supplementary Table S3. miR-185 expression in esophageal cancers.

miR-185 expression	Affected gene / pathway	Functional consequences	Samples analyzed	Ref.
Down	*KLK5*	Upregulation reduces malignant behavior of cells and leads to better prognosis	- Tissue (n=57)<br>- Cell lines KYSE-30, TE-1, Eca-109, EC9706, and KYSE-150<br>- Animal tissue	[36]
Down	*RAGE/HSP27*	Suppression of proliferation, migration, and invasion	- Tissue (n=29)<br>- Plasma (n=28)<br>- Cell lines Eca-109, TE-11<br>- Animal tissue	[37]
Down	*SIX1*	Inhibition of proliferation, migration, and invasion	- Tissue (n= 23)<br>- Cell lines KYSE150 and KYSE30	[38]
Up	*KLF3*	Increased invasion, proliferation, migration, and tumor size	- Cell line Eca-109<br>- Animal tissue	[44]
Up	-	Upregulation is a potential diagnostic biomarker and is associated with a good prognosis.	- Tissue (n= 38)	[33]

Supplementary Table S4. miR-185 expression in gastric cancers.

miR-185 expression	Affected gene / pathway	Functional consequences	Samples analyzed	Ref.
Down	*ARC*	Contributor to chemosensitivity	- Tissue (n=25)<br>- Cell lines NCI-N87, MGC-803, BGC-823, and AGS<br>- Animal tissue	[54]
Down	*BCL-2, CASP-3, CASP-8, XIAP*	Associated with clinicopathological features and enhanced apoptosis	- Tissue (n=30)<br>- Cell lines MKN74, SGC7901, BGC823, and MGC803	[58]
Down	*BCL-2, MDR1/P-gp, MRP-1*	Involvement in chemoresistance	- Tissue (n=70)<br>- Cell lines SGC7901, GES-1, and SGC7901/ADR	[55]
Down	*CCND2*	Decreased apatinib-resistance	- Tissue (n=80)<br>- Cell line MGC-803/AP	[68]
Down	*CTSD*	Overexpression leads to inhibition of EMT and migration and augmentation of apoptosis and cell cycle arrest	- Tissue (n=10)<br>- Cell lines MKN-1, AGS, SGC7901, NCI-N87, BGC823	[60]
Down	*DNMT1*	Correlation with metastasis and poor prognosis	- Tissue (n=162)<br>- Cell lines MGC-803, BGC-823, MKN-28, SGC-7901, HGC-27, AGS, and MKN-45<br>- Animal tissue	[57]
Down	*DNMT1, EZH2*	Associated with methylation status	- Tissue (n=24)	[48]
Down	*DNMT1, EZH2*	Inhibition of proliferation and arrest of cell cycle	- Tissue (n=80)<br>- Cell lines AGS, MKN1, and MKN28	[49]
Down	*DNMT1, EZH2*	Upregulation mediates NH_2-terminal hydrophobic region and BRICHOS domain of the GKN1 tumor suppressive effects	- Cell line AGS	[50]
Down	*MDR1/P-gp, MRP-1, GST-π*	Inhibition improves resistance to chemotherapeutic drugs	- Tissue (n=25)<br>- Cell lines SGC7901 and SGC7901/ADR	[53]
Down	*RHOA*	Decreased migration and invasion	- Tissue (n=35)<br>- Cell lines AGS and MKN1	[51]
Down	*TGF-β1*	Decreased cell growth, migration, and invasion	- Cell lines MGC803, BGC823, SGC-7901, AGS, KATOIII, and HEK-293T	[67]
Down	*TRIM29*, Wnt/β-catenin pathway	Induction of apoptosis, reduction of cell proliferation, arrest of cell cycle	- Cell lines MGC803 and BGC823	[59]
Down	-	Prediction of effectiveness of chemotherapy	- Tissue (n=120)	[56]

Up	Putative targets: *AQP5, ESRRA, RAC3, RGS14, ZNFN1A4*	Involved in development and progression	- Tissue (n=10)	[61]
Up	-	Expression associated with EBV-infected and uninfected cancers	- Tissue (n=100)	[62]
Up	-	Diagnosis	- Tissue (n=216)<br>- Serum (n=370)<br>- Pooled serum (n=4)<br>- Exosomes in serum (n=58)	[65]
Up	-	Diagnosis	- Tissue (n=60)<br>- Plasma (n=192)<br>- Pooled plasma (n=4)<br>- Arterial plasma (n=38)<br>- Exosomes in plasma (n=20),	[64]

Supplementary Table S5. miR-185 expression in hepatocellular carcinoma (HCC).

miR-185 expression	Affected gene / pathway	Functional consequences	Samples analyzed	Ref.
Down	*AKT*	Decreased proliferation, invasion, and migration and increased apoptosis	- Cell lines HepG2, Huh-7, SMMC-7721, Bel-7402, and Hep3B	[84]
Down	Akt1 signaling pathway	Induction of autophagy and apoptosis and inhibition of cell cycle	- Cell line HepG2	[77]
Down	*AQP11, KIF14, KNL1, MKI67, SPC25*	Arrested cell cycle and increased necroptosis	- Cell lines HepG2, Huh-7, Hep3B, HLE, HLF, HeLa, and HEK293	[85]
Down	*CDC42*	High expression associated with good prognosis as well as suppressed migration and invasion through downregulation of CDC42	- Tissue (n=63)<br>- Cell line Huh-7	[71]
Down	*CDH1, SIX2, VIM*	Suppressed cell growth, metastasis, and EMT progression	- Cell lines HepG2, Huh-7, SNU-387, and SNU-449	[74]
Down	*DNMT1*	Overexpression decreases the expression of DNMT1	- Cell lines Huh-7 and TFK-1	[76]
Down	*DNMT1, DNMT3A, DNMT3B, MEG3*	Increased expression of tumor suppressive gene MEG3	- Cell lines HepG2 and Huh-7	[87]
Down	DNMT1/PTEN/Akt pathway	Decreased cell proliferation and invasion	- Tissue (n=40)<br>- Cell lines HepG2, Huh7, and Hep3B cells<br>- Animal tissue	[75]
Down	*ELK1*	Decreased HBV proteins through suppression of ELK1	- Cell lines Huh-7 and HepG2.2.15	[89]
Down	ITGB5/β-catenin pathway	Low expression correlated to tumorigenesis through activation of ITGB5/β-catenin pathway	- Tissue (n=61)<br>- Cell lines Huh-7 and MHCC-97L<br>- Animal tissue	[73]
Down	*ROCK2*	Reduced invasion and migration	- Tissue (n=44)<br>- Cell lines Hep-3B and SNU-387	[72]
Down	-	Reduced miR function following RACK1-knockdown	- Tissue (n=3)<br>- Cell lines Huh-7, PLC/PRF/5, and 293T	[88]
Down	-	Potential serum biomarker for HCV-related HCC	- Serum (n=40)	[80]
Down	-	Associated with survival, recurrence, cell growth, and invasion	- Tissue (n=95)	[78]
Up	PLAC8/Wnt/ β-catenin pathway	Increased HCC progression	- Tissue (n=30)<br>- Cell lines Huh-7, Hep3B, and HepG2<br>- Animal tissue	[83]
Up	-	Discrimination of HBV-positive HCC patients from HBV-positive cancer-free controls	- Plasma (n=67)	[81]
Up	-	Associated with venous metastasis	- Tissue (n=482)	[82]

Supplementary Table S6. miR-185 expression in pancreatic cancer (PDAC).

miR-185 expression	Affected gene / pathway	Functional consequences	Samples analyzed	Ref.
Down	*CBX2*	Overexpression inhibits proliferation, migration and invasion	- Tissue (n=67)<br>- Cell lines Capan-2, AsPC-1, PANC1, BxPC-3, and HPDE	[91]
Down	*CCND2*	Decreased cell proliferation, arrested cell cycle, promoted apoptosis	- Tissue (n=70)<br>- Cell lines PANC-1, ASPC-1, HPAC, and BxPC-3	[90]
Down	*TAFAZZIN*	Decreased TAZ expression and cell proliferation	- Tissue (n=46)<br>- Cell lines HPAC, PANC-1, and 293T<br>- Serum (n=46)<br>- Pancreatic fluid (n=46)	[93]
Up	*CORO2B, NTRK3*		- Tissue (n=32; database records)	[94]
Up	-	Potential diagnostic and prognostic biomarker	- Serum (n=197)	[95]

Supplementary Table S7. miR-185 expression in colorectal cancer (CRC).

miR-185 expression	Affected gene / pathway	Functional consequences	Samples analyzed	Ref.
Down	*AQP5*	Increased chemosensitivity	- Tissue (n=120)<br>- Cell lines HCT-116 and HCT-8	[103]
Down	*CDC42*	High expression reduces proliferation, migration, and invasion	- Tissue (n=25)<br>- Cell lines SW620, HCT-116, HT-29, and LOVO	[126]
Down	*CDC42, RHOA*	Decreased proliferation and invasion and increased cell cycle arrest and apoptosis	- Cell lines SW1116 and LOVO	[101]
Down	*CDK4, CDK6*	Decrease of proliferation and increase of apoptosis	- Tissue (n=25)<br>- Cell lines HCT-116, HCT-8, HT-29, DLD-1, SW620, and FHC<br>- Animal tissue	[127]
Down	*c-MYC*	Low expression associated with enhanced c-Myc and cell proliferation	- Cell line HCT-116	[102]
Down	*DC-SIGN*	Decreased metastasis	- Tissue (n=56)<br>- Cell lines LS174T, HCT116, LOVO, SW620, SW480, and HT29<br>- Animal tissue	[107]
Down	*FSCN1*	Suppression of FSCN1 and inhibition of tumor progression	- Tissue (n=94)<br>- Cell lines HCT116, SW480, HT29, NCM460, SW620, and CaCO2<br>- Animal tissue	[124]
Down	*HIF-2α*	Reduced proliferation and invasion	- Tissue (n=30)<br>- Cell lines LS174T, HCT116, LOVO, SW620, SW480, and HT29<br>- Animal tissue	[134]
Down	*HMGA2*	Reduced proliferation, migration, invasion and cell cycle progression and increased apoptosis	- Tissue (n=50)<br>- Cell lines LoVo, SW620, SW480, and HT29	[129]
Down	*IGF1R, IGF2*	Enhancement of radiosensitivity	- Cell line RR−HCT116	[105]
Down	*IGF2*	Decreased invasion and migration via IGF2 suppression	- Tissue (n=10)<br>- Cell lines SW620 HT-29, HCT 116, LOVO, and SW480	[125]
Down	*MAPK14*, MAPKAPK2/HSP27 pathway	Overexpression of miR-185 targets MAPK14 and inactivates MAPKAPK2/HSP27 pathway	- Cell lines SW480, SW620, and HT-29<br>- Animal tissue	[122]
Down	*NRP1*	Decreased metastasis	- Tissue (n=284)	[109]

			- Cell lines Caco2, HCT116, HT29, LoVo, RKO, SW1116, SW480, NCM460, and HEK 293T<br>- Animal tissue	
Down	*NOTCH3*	Decreased proliferation and metastasis	- Cell lines DLD-1, SW480, SW48, HEK-293T, and HCT-116	[123]
Down	*STIM1*	Decreased migration and invasion and reversed EMT	- Tissue (n=40)<br>- Cell lines SW480, SW620, HT29, and LOVO<br>- Animal tissue	[104]
Down	*TEAD1*	Mitigated proliferation, migration, and invasion	- Cell lines	[130]
Down	WISP2/β-catenin pathway	Inhibition of proliferation and induction of autophagy	- Cell lines SW620 and HT29<br>- Animal tissue	[100]
Down	Wnt1/β-catenin pathway	Upregulation of miR-185 targets Wnt1 and restrains Wnt1/β-catenin pathway	- Tissue (n=41)<br>- Cell lines HCT-116, LS174T, and SW480	[98]
Down	Wnt/β-catenin pathway	Decreased tumorigenesis	- Cell lines LOVO and SW480	[99]
Down	*YY1*	Inhibition of metastasis and stemness	- Tissue (n=30)<br>- Cell lines SW480, HCT116, SW620 and HT-29), animal	[128]
Down	-	Expression correlated with advanced clinical stage and metastasis	- Tissue (n=52)	[111]
Down	-	Decreased in patients with recurrence of liver metastasis	- Serum (n=20)	[114]
Down	-	Expression has prognostic value	- Tissue (n=85)	[113]
Up	*ARID1A*	Downregulation of *ARID1A*	- Tissue (n=258)<br>- Cell lines HCT116 and LoVo	[119]
Up	-	Expression related to carcinogenesis in colon CSCs	- Cell line HT-29	[120]
Up	-	Overexpression related to poor survival and metastasis	- Tissue (n=50)	[118]
Up	-	Upregulation during MNNG induced carcinogenesis	- Cell line IEC-6	[121]
